# Transmission-blocking Effects of Primaquine and Methylene Blue Suggest *Plasmodium falciparum* Gametocyte Sterilization Rather Than Effects on Sex Ratio

**DOI:** 10.1093/cid/ciz134

**Published:** 2019-02-12

**Authors:** John Bradley, Harouna M Soumaré, Almahamoudou Mahamar, Halimatou Diawara, Michelle Roh, Michael Delves, Chris Drakeley, Thomas S Churcher, Alassane Dicko, Roly Gosling, Teun Bousema

**Affiliations:** 1 Medical Research Council (MRC) Tropical Epidemiology Group, London School of Hygiene and Tropical Medicine, United Kingdom; 2 Malaria Research and Training Centre, Faculty of Pharmacy and Faculty of Medicine and Dentistry, University of Science, Techniques and Technologies of Bamako, Mali; 3 Global Health Group, Malaria Elimination Initiative, University of California, San Francisco; 4 Department of Immunology and Infection, London School of Hygiene and Tropical Medicine, United Kingdom; 5 MRC Centre for Global Infectious Disease Analysis, Imperial College London, United Kingdom; 6 Radboud Institute for Health Sciences, Radboud University Medical Center, Nijmegen, The Netherlands

**Keywords:** malaria, infectiousness, anopheles, transmission, treatment

## Abstract

Gametocyte density and sex ratio can predict the proportion of mosquitoes that will become infected after feeding on blood of patients receiving nongametocytocidal drugs. Because primaquine and methylene blue sterilize gametocytes before affecting their density and sex ratio, mosquito feeding experiments are required to demonstrate their early transmission-blocking effects.

Transmission of malaria depends on mature sexual stage parasites (gametocytes) in the peripheral blood. Blood-feeding *Anopheles* mosquitoes must ingest at least 1 male and 1 female gametocyte to become infected. The formation of *Plasmodium* oocysts on the mosquito midgut wall is commonly used as evidence for successful transmission. These oocysts enlarge over time to rupture and release sporozoites that render mosquitoes infectious. In the absence of treatment, infectivity of malaria-infected individuals can be reasonably well predicted by the density of male and female gametocytes in their peripheral blood [1]. Antimalarial drugs differ in their activity against gametocytes [2]. Artemisinins effectively clear asexual parasites and developing gametocytes but have limited efficacy against mature gametocytes [3–5] and therefore do not fully prevent transmission after treatment [4].

Primaquine (PQ) and methylene blue (MB) are potent gametocytocidal compounds. For these drugs, effects on transmissibility may occur before a measurable impact on total gametocyte density [6]. One plausible explanation for this early transmission blockade would be a preferential clearance of male or female gametocytes, resulting in a distorted, nonviable gametocyte sex ratio [7]. Gametocytocidal drugs may indeed exert different effects on male and female gametocytes [2, 8], with MB preferentially clearing male gametocytes [4] and indications that PQ may preferentially clear female gametocytes [4]. An alternative explanation for early transmission blockade would be a gametocyte-sterilizing effect, that is, a reduction in gametocyte infectivity in the absence of, or prior to, an effect on density. We recently presented a mathematical model to predict mosquito infection rates based on gametocyte density and sex ratio [1]. Here, we utilize this model to determine whether early transmission-blocking effects of PQ and MB can be explained by the effects these compounds have on gametocyte density or sex ratio or whether there is evidence for a sterilizing effect that precedes gametocyte clearance.

## METHODS

We used data from a previously reported, single-blind, randomized, controlled trial that received ethical approval from the University of Science, Techniques, and Technologies (Bamako, Mali), the University of California–San Francisco, and the London School of Hygiene & Tropical Medicine (United Kingdom) [4]. The trial compared sulfadoxine-pyrimethamine and amodiaquine (SP/AQ) and dihydroartemisinin-piperaquine (DP) as nongametocytocidal drugs with no established effects on mature gametocytes, with SP/AQ plus a single dose of 0·25 mg/kg PQ (SP/AQ-PQ) and DP plus 15 mg/kg MB per day for 3 days (DP-MB) as gametocytocidal drug combinations [4]. Following written informed consent (participants aged ≥18 years) or parental consent (participants aged <18 years) with assent for children aged 12–17 years, asymptomatic microscopy-positive gametocyte carriers aged 5–50 years were recruited in Ouélessébougou, Mali. Infectivity of participants to mosquitoes (the percentage of mosquitoes that develop oocysts) was assessed by membrane feeding assay prior to treatment and on days 2 and 7 after initiation of treatment. Density of male and female gametocytes was determined by quantitative reverse-transcription polymerase chain reaction assays that target female (Pfs25) and male (PfMGET) gametocyte transcripts with sex-specific trend lines [9]. Comparisons of gametocyte densities were done using *t* tests on log-transformed values, giving estimates of the ratio of geometric mean (RGM) densities of male and female gametocytes between treatment groups. To make log-transformation possible, zeros were imputed to 0.005 gametocytes per microliter (half the minimum detectible concentration). Infectivity in DP-MB and SP/AQ-PQ arms was compared with that of nongametocytocidal drugs (DP or SP/AQ) using a previously published statistical model, controlling for male and female gametocyte density [1]. Because of low infectivity following DP-MB or SP/AQ-PQ, it was not possible to estimate the relative reduction in probability of transmission conditional upon gametocyte density (models failed to converge). To overcome this, the nonparametric van Elteren’s test, an extension of the Wilcoxon rank sum test that compares groups in a stratified manner [10], was used. Because gametocyte density is a strong nonlinear determinant of infectivity, we compared infectivity between groups after stratifying in categories 1, 1 to <5, 5 to <20, 20 to <50, 50 to <100, and ≥100 total gametocytes per microliter. Infectivity was then compared within different bins to test for differences between arms, accounting for gametocytemia.

## RESULTS

**Figure 1.**
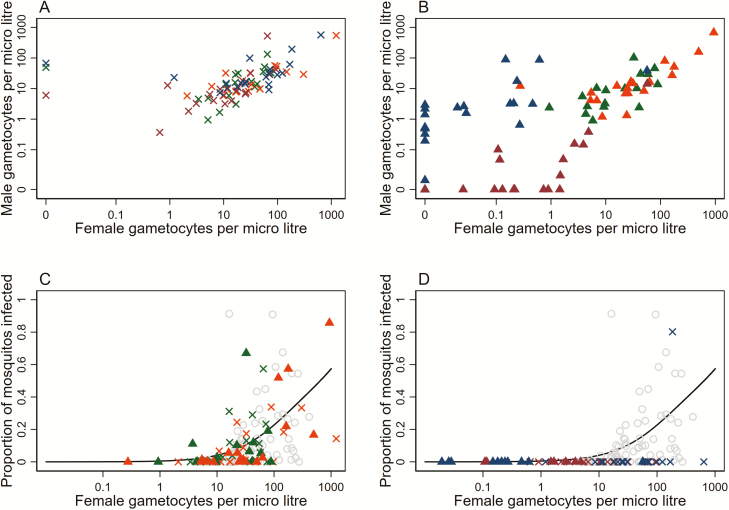
Associations between gametocyte density, sex ratio, treatment, and the proportion of mosquitoes that developed oocysts (became infected). The association between male and female gametocyte density is presented for day 2 (*A*) and day 7 (*B*) post-initiation of treatment. The association between female gametocyte density and the proportion of infected mosquitoes is presented for individuals who received nongametocytocidal drugs (*C*) and gametocytocidal drugs (*D*). In all panels, green symbols represent observations following dihydroartemisinin-piperaquine (DP); orange, sulfadoxine-pyrimethamine and amodiaquine (SP/AQ); maroon, DP-methylene blue; and blue, SP/AQ-primaquine. Crosses represent feeding outcomes from experiments conducted on day 2 post-initiation of treatment and triangles represent feeding outcomes from experiments conducted on day 7 post-initiation of treatment. (*C* and *D*) The proportion of mosquitoes that developed oocysts prior to treatment plotted in light gray circles for all treatment arms for illustrative purposes; the black line indicates the shape of the best fit relationship between female gametocyte density and infectivity in the absence of malaria treatment as previously defined [1].

The study was conducted from 27 June 2016 through 1 November 2016. Samples from 80 participants were analyzed at enrollment and on day 2 and day 7 post-initiation of treatment. On day 2 there was no evidence that female gametocyte densities were lower following treatment with DP-MB (RGM with 95% confidence interval 0.37 [0.12, 1.13]; *P* = .079) or SP/AQ-PQ (RGM = 1.23 [0.39, 3.94]; *P* = .718) compared to nongametocytocidal drugs (Figure 1A). Similarly, there was no evidence that male gametocyte densities were lower on day 2 following treatment with DP-MB (RGM = 0.54 [0.26, 1.09]; *P* = .085) or SP/AQ-PQ (RGM = 1.81 [0.95, 3.46]; *P* = .071) compared to nongametocytocidal drugs (Figure 1A). In contrast, treatment with DP-MB reduced day 7 densities of both female (RGM = 0.015 [0.004, 0.053]; *P* < .001) and male gametocytes (RGM = 0.002 [0.001, 0.007]; *P* < .001) and treatment with SP/AQ-PQ reduced day 7 densities of both female (RGM = 0.003 [0.001, 0.010]; *P* < .001) and male gametocytes (RGM = 0.254 [0.082, 0.787]; *P* = .018) compared to nongametocytocidal drugs (Figure 1B).

In a model adjusted for male and female gametocyte density, there was no evidence that among nongametocytocidal arms infectivity differed between baseline and day 2 (risk ratio [RR] = 1.08 [0.57, 2.03]; *P* = .819; Figure 1C) and between baseline and day 7 (RR = 1.51 [0.71, 3.32]; *P* = .288; Figure 1C). Infectivity at a given density was similar for those on SP-AQ compared to DP (RR = 0.72 [0.36, 1.43]; *P* = .345). Despite considerable overlap of gametocyte densities in all arms, infectivity after DP-MB and SP/AQ-PQ was substantially lower on day 2. For those on nongametocytocidal drugs, 22/36 (61%) were infectious to mosquitoes on day 2 compared to 0/19 (0%) on DP-MB and 1/19 (5%) on SP/AQ-PQ, with strong evidence for lower infectivity after adjusting for gametocyte density (van Elteren’s test, *P* < .001). The reduction in infectivity on day 2 was thus not explained by gametocyte density (Figure 1D) or sex ratio (Figure 1A). Infectivity for gametocyte-positive individuals on MB and PQ remained lower on day 7, with 19/36 (53%) on nongametocytocidal drugs infectious to mosquitoes compared to 0/8 (0%) of those on MB and 0/11 (0%) of those on PQ (Figure 1D). There was less overlap in gametocyte densities between those on MB or PQ and those on gametocytocidal drugs. However, again there was evidence that infectivity was lower for those on gametocytocidal drugs after adjusting for gametocyte density (van Elteren’s test, *P* = .044).

## DISCUSSION

We found that measures of mRNA gametocyte density do not correlate with infectivity shortly after treatment with gametocytocidal drugs. The major implication of this finding is that studies that examine transmission-blocking effects of drugs and potentially vaccines require mosquito-feeding assays to measure infectivity. Gametocytocidal drugs rapidly clear gametocytes, distort the gametocyte sex ratio [4, 11], and prevent onward transmission to mosquitoes [4, 6, 7]. Although it was previously reported that the transmission-blocking effect of PQ may precede its gametocyte clearing effect [7], it has been unclear to what extent an early effect of sex ratio may contribute to this rapid prevention of onward transmission [7]. For MB we previously hypothesized that its preferential clearance of male gametocytes may (partially) explain its early transmission-blocking effects [4].

Here, we present evidence that gametocyte density and sex ratio are unaffected in the first 48 hours after initiation of treatment, despite a near complete prevention of onward transmission to mosquitoes. Nongametocytocidal drugs did not alter the shape of the associations between gametocyte density, sex ratio, and mosquito infection prevalence; gametocytes appeared equally infectious before and after treatment. In contrast, gametocyte density and sex ratio no longer explained transmission after treatment with DP-MB or SP/AQ-PQ; gametocytes persisted with sex ratios similar to those before treatment, but mosquito infections were nearly completely prevented.

The mechanism that underlies the early sterilizing effect despite continued presence of gametocyte mRNA transcripts is unclear. While mRNA is unlikely to survive in free-floating form and, also by microscopy, gametocyte densities remain unaffected shortly after treatment [6], it is possible that mRNA persists in red blood cells with parasites that are damaged by treatment. Upon reaching maturity, gametocytes complete their preparations for onward development and maintain cell cycle arrest until triggered to differentiate into gametes in the mosquito. Therefore, metabolic activity of mature gametocytes is likely reduced to “housekeeping” functions such as ATP production and general redox activity. Antimalarials that cause generalized cellular damage such as oxidative stress induced by MB inhibition of glutathione reductase [12] may manifest an initial transmission-blocking effect that is only apparent when gametocytes re-engage the complex cellular processes required for successful gametogenesis. PQ is similarly postulated to operate through reactive metabolites that cause nonspecific damage to gametocytes [13].

The molecular mechanisms that underlie the apparent male-biased clearance of gametocytes after MB treatment and female-biased clearance after PQ treatment are currently unclear; however, they likely reflect the sexual dimorphism of gametocyte proteomes. Our findings thus provide evidence for an early sterilizing effect of MB and PQ. Molecular tools to quantify male and female gametocytes cannot replace mosquito-feeding experiments to assess transmission-blocking properties of antimalarials.

## Notes

***Financial support.***The original clinical trial was supported by a grant from the Bill & Melinda Gates Foundation (OPP1089413) and a fellowship from the European Research Council (ERC-2014-StG639776). J. B., C. D., and T. B. are further supported by a grant from the Bill & Melinda Gates Foundation (INDIE OPP1173572).

***Potential conflicts of interest.***T. B. reports salary support from the European Research Council (ERC-2014-StG639776) and support from the Bill & Melinda Gates Foundation (OPP1173572). R. G. reports salary support from the Bill and Melinda Gates Foundation (OPP1089413) and the Agency for International Development outside the submitted work. All other authors report no potential conflicts. All authors have submitted the ICMJE Form for Disclosure of Potential Conflicts of Interest. Conflicts that the editors consider relevant to the content of the manuscript have been disclosed.
